# Cerebrospinal Fluid Proteins as Biomarkers of Brain Vascular Injury

**DOI:** 10.1002/alz70856_107687

**Published:** 2026-01-09

**Authors:** Antoine R Trammell, Aliza P. Wingo, Monica W Parker, Felicia C. Goldstein, James J. Lah, Amy D. Rodriguez, Rebecca McIntosh, Thomas S. Wingo

**Affiliations:** ^1^ Emory University Goizueta Alzheimer's Disease Research Center, Atlanta, GA, USA; ^2^ University of California, Davis, Sacramento, CA, USA; ^3^ Emory University School of Medicine, Atlanta, GA, USA; ^4^ Goizueta Alzheimer's Disease Research Center, Emory University, Atlanta, GA, USA

## Abstract

**Background:**

Mounting evidence shows relationships between the cerebrovasculature, cognitive decline, neurodegeneration, and amyloid accumulation that underscores the contribution of cerebrovascular atherosclerotic disease (CA) to the pathogenesis of Alzheimer's disease and related dementias (AD/ADRD). Previously, we identified 114 brain proteins associated with CA as measured by carotid artery intimal media thickness (IMT). That finding suggests that surrogate brain biomarkers of CA may hold promise as sensitive markers for early ADRD detection. Compared to brain tissue, cerebrospinal fluid (CSF) is more accessible and can be collected from living persons. Thus, among the brain proteins profiled, we also profiled 6 (CBR1|P16152, ENDOD1|O94919, MOG|Q16653. PCSK1|P29120, PLP1|P60201, and QDPR|P09417) in CSF. In this follow‐up study, we explored the CSF proteome for associations between profiled proteins and CA as measured by carotid artery IMT. Hence, this study aims to explore potential biomarkers of CA and ADRD risk by examining associations between CSF proteins and IMT.

**Method:**

All CSF samples and vascular measurements were collected from Emory Goizueta Alzheimer's Disease Research Center research participants. We described the CSF collection steps and multiplex proteomic steps previously.^7^ We performed proteomic analysis on 86 CSF samples, followed by linear regression adjusted for age, sex, and cognitive diagnosis (control vs. impaired) to test associations with CA. We applied BoxCox transformation to IMT measures to improve normality and we excluded participants with AD.

**Result:**

Data for 83 people were analyzed. The sample's mean age was 65.8 (8.02); 60% were female, 70% were white adults, and 69% were controls. Among white participants, QDPR|P09417 (neurotransmitter production) was associated with higher IMT. For AA participants, PLP1|P60201 (myelin sheath) was associated with higher IMT. In meta‐analysis (both groups), QDPR|P09417 and MOG|Q16653 (myelin sheath) were associated with higher mean carotid IMT.

**Conclusion:**

Vascular disease can co‐occur with AD. Our results show associations between CSF proteins involved in structural integrity and chemical signaling and CA in a sample with impaired and normal cognition. Further, we detected racial differences in these associations. Given these findings among cognitively normal and impaired people, these proteins may have promise as early disease indicators. More extensive study with a larger sample is needed.

